# Rapid Screening Method to Assess Formation Damage During Injection of Metal Oxide Nanoparticles in Sandstone

**DOI:** 10.3390/nano16070402

**Published:** 2026-03-26

**Authors:** Craig Klevan, Bonnie A. Marion, Jae Jin Han, Taeyoung Chang, Shuhao Liu, Keith P. Johnston, Linda M. Abriola, Kurt D. Pennell

**Affiliations:** 1 School of Engineering, Brown University, Providence, RI 02912, USA; craig.klevan@geosyntec.com (C.K.); linda_abriola@brown.edu (L.M.A.); 2 Department of Civil and Environmental Engineering, Tufts University, Medford, MA 02155, USA; bmarion@ftdsolutions.net (B.A.M.);; 3 McKetta Department of Chemical Engineering, The University of Texas at Austin, Austin, TX 78712, USA; taeyoung@utexas.edu (T.C.); kpj@che.utexas.edu (K.P.J.); 4 The Jasper Department of Chemical Engineering, The University of Texas at Tyler, Tyler, TX 75799, USA; sliu@uttyler.edu

**Keywords:** nanoparticles, sandstone, face-caking, formation damage, enhanced oil recovery (EOR)

## Abstract

Many advances in enhanced oil recovery (EOR) take advantage of the unique properties of nanomaterials to improve characterization of formation properties, achieve conformance control during flood operations, and extend the controlled release time of polymers. Magnetite nanoparticles (nMag) have been employed in these processes due to their low cost, low toxicity, and ability to be engineered to meet desired needs, especially with the application of a magnetic field. Similarly, silica dioxide (SiO_2_) and aluminum oxide (Al_2_O_3_) nanoparticles have been evaluated for the delivery of scale and asphaltene inhibitors. However, the injection of nanoparticles into porous media comes with the risk of formation damage due to particle deposition, which can lead to increased injection pressures and reductions in permeability. The goal of this study was to develop a method to evaluate and assess nanoparticle formulations for their potential to cause formation damage. A screening apparatus was constructed to hold small sandstone discs (~2 mm) or cores (~2.5 cm) for rapid testing with minimal material use and the capability to be used with either aqueous brine solutions or non-polar solvents as the mobile phase. Image analysis of the disc and pressure measurements demonstrated increasing deposition of nMag and face-caking when the salinity was increased from 500 mg/L NaCl (8.56 mM) to API brine (2.0 M). Similarly, when the injected concentration of silica nanoparticles in 500 mg/L NaCl was increased from 1 to 10 wt%, the back pressure increased by 55 psi, and face-caking was observed. The screening test results were consistent with traditional core-flood tests and was able to be modified to accommodate organic liquid mobile phases. The screening test results closely matched nanoparticle transport and retention measured in sandstone cores, confirming the ability of the system to rapidly screen nanoparticle formulations for potential formation damage.

## 1. Introduction

Fossil fuels continue to account for the majority of energy consumption in the world [1], and the current demand for fossil fuels is likely to persist for many decades, based on global trends for energy usage [2]. Since oil is a finite resource, it is important to explore methods that are able to improve the extraction of oil from reservoirs, as it is possible that several trillion barrels of oil would remain in reservoirs if only conventional methods are applied for oil extraction [3]. Conventional extraction of oil typically involves the injection of large quantities of water into the reservoir formation to displace the trapped oil. Since water displacement may not be uniform (i.e., bypassing) and residual oil will be trapped in the formation due to capillary forces, the recovery efficiency can be as low as one-third of the total oil, and may decrease even further due to other factors such as oil viscosity, subsurface heterogeneity, and reservoir wettability [4,5]. Furthermore, internal reservoir processes such as solid precipitation (e.g., mineral scale or asphaltenes) and equipment fouling (e.g., scale growth and corrosion) lead to decreases in oil production, leading to investigations into solutions to prevent these seemingly unavoidable issues [6,7,8].

The most common methods for enhanced oil recovery (EOR) involve the injection of chemical additives into the reservoir formation to alter the wettability of the formation, reduce interfacial tension at oil–water interfaces, or improve conformance control by altering permeability, which in turn allows for larger quantities of oil to be recovered during the water displacement process [9]. The chemicals used include surfactants or engineered polymers designed to withstand harsh conditions that are typically encountered during oil extraction (e.g., high salinities and temperature) [10,11]. Recent advances in EOR technology incorporate the injection of engineered nanoparticles to further improve recoveries. For example, a study comparing oil recovery achieved by using polymer alone with that using both polymer and nanoparticles reported an almost 20% increase in oil recovery with the addition of nanoparticles [12]. In EOR, metal oxide nanoparticles (e.g., AlO_3_, Fe_2_O_3_, SiO_2_) have been shown to improve oil recovery through mechanisms such as lowered oil viscosity, reduced interfacial tension between water and oil, or favourably altering the wettability of the reservoir minerals [13,14,15,16,17]. Nanoparticles may also be used to deliver oligomers and polymers that are slowly released to mitigate the formation of mineral scales and asphaltene deposits, which may clog pores, decreasing oil production [18,19,20,21].

In practice, nanoparticles are typically coated with polymers to improve suspension stability and to impart specific properties to meet the requirements of EOR applications [22,23]. For example, iron oxide nanoparticles (IONPs) coated with cetyltrimethylammonium bromide (CTAB) have been found to significantly increase oil recovery through wettability modification and prevention of scale formation [24]. Electromagnetic sensing with magnetite nanoparticles (nMag) can support greater resolution for reservoir characterization and monitoring [25,26,27,28]. Silica nanoparticles coated with hydrophilic polymers were shown to increase oil recovery through reductions in the oil–water interfacial tension, decreased reservoir surface roughness, and nanoparticle stabilization of large oil droplets to reduce pore clogging [29]. The nanoparticle type and concentration, as well as dispersing fluid and co-constituents, such as surfactants, are all important variables that can be modified or adjusted to improve performance and the degree of nanoparticle transport through the reservoir [17,29,30,31].

When injecting nanoparticles into a consolidated oil reservoir formation, the particles should exhibit high mobility with minimal effects on formation permeability and integrity [32]. Although various types of nanoparticles and nanoparticle–surfactant combinations have been shown to greatly improve oil recovery, the use of nanoparticles presents a potential risk of decreasing the reservoir permeability due to processes such as particle aggregation, sedimentation, and attachment to mineral surfaces, all of which may contribute to pore clogging [33,34]. In general, a greater concentration of nanoparticles leads to increased oil recovery, as more nanoparticle attachment leads to increased wettability alteration, which is favourable to oil flow [35]. However, this relationship typically exhibits a threshold concentration above which the injected nanoparticle concentration is too high and instead causes a decrease in oil recovery as the retained particles block reservoir pore throats rather than uniformly coating the mineral surfaces [36,37]. If the nanoparticle formulations are not stable over the duration of the injection, aggregation of nanoparticles may also occur [34].

The phenomena of nanoparticle pore clogging and blocking are most likely to occur near the point of injection into the formation [37,38]. The deposition of nanoparticles near the injection point in the formation can cause the accumulation of deposited particles to form into a solid ‘cake’, in a process commonly referred to as “face-caking” due to pore blockage and corresponding increases in injection pressure [39]. The associated undesirable reduction in reservoir permeability is referred to as formation damage, which can lead to increased costs in terms of decreased oil recovery and production cessation [40]. Formation damage tests have been developed to evaluate potential formation permeability reduction risks attributed to factors such as the swelling of internal clay minerals [41]. For nanofluids (liquids containing nanoparticles) injection, screening approaches have been developed to compare interfacial tension reducing properties and oil recovery, and computational fluid dynamics (CFD) models have been used to assist in determining the best nanoparticle sizes for EOR applications [42,43]. Nanoparticles are typically evaluated for their ability to improve oil recovery using core flood tests, which are labor-intensive and expensive. A group studying polymer-coated silica nanoparticles in core flood tests for EOR observed increases in oil recovery with several samples, yet also performed experiments with samples that aggregated and caused the formation of a visible cake on the sandstone core [29]. As nanoparticles and other EOR compounds may exist in numerous combinations in various injection fluids, a rapid screening test for formation damage caused by nanoparticle caking could prove useful in facilitating the selection of nanoparticle formulations for EOR applications. Existing approaches for evaluating nanoparticle-induced formation damage may rely on numerical simulations or filtration-based tests [44,45]. Modelling methods such as numerical approaches and computational fluid dynamics (CFD) have been used to screen nanoparticle sizes and transport [42,46,47]; however, these approaches often require simplifying assumptions regarding pore geometry and particle interactions and do not capture face-caking directly under experimental flow conditions. Likewise, simple filtration or membrane-based tests can indicate particle plugging tendencies but offer little perspective regarding the behaviours of the reservoir rock.

Due to the potential for formation damage during nanoparticle delivery, the goal of this study was to develop and validate a rapid (<1 h) experimental method to identify nanoparticle formulations or conditions that could result in face-caking and formation permeability reductions. A flow-through sandstone disc holder was developed to quickly test nanoparticle formulations for formation damage. Visual observations and image analysis of face-caking and injection-pressure were used to assess the ability of the disc holder to screen nanoparticles for potential formation damage effects. The system was modified to allow for testing with both aqueous brines and organic solvents as the mobile phase. In contrast to other approaches, the disc-based screening method developed in this study preserves the native sandstone pore geometry and mineral surface properties while enabling rapid evaluation of nanoparticle formulations under controlled flow conditions. The approach allows direct visualization of the face-caking at the inlet surface, quantitative monitoring of pressure evolution, and assessment of permeability reduction using minimal material and time compared to full core-flood experiments. As such, the method provides an experimentally grounded, rapid screening platform for the down selection of nanoparticle formulations. The rapid screening method was tested for iron oxide, silica oxide, and aluminum oxide nanoparticle formulations, and compared to transport and retention data obtained for longer cores with and without the addition of polymers used to enhance nanoparticle mobility.

## 2. Experimental Materials and Methods

### 2.1. Materials

Nanoparticle mobility and formation damage experiments were conducted with Berea sandstone (permeability: 223 mD; porosity: 23%), as this material is most often used in EOR studies. Sandstone cores were obtained from Kocurek Industries (Caldwell, TX, USA) and cut to 2.54 cm diameter × 7.5 cm length by Core Laboratories Inc. (Duncan, OK, USA). Iron (II) acetate (Fe(C_2_H_3_O_2_)_2_) (purity: ≥95%) and triethylene glycol (TEG) (purity: ≥99%) used for nanoparticle synthesis were purchased from Sigma Aldrich (St. Louis, MO, USA). Sodium chloride (purity: ≥99%) and calcium chloride (purity: ≥96%) were purchased from Fisher Scientific (Waltham, MA, USA) and were used to prepare American Petroleum Institute (API) brine (8% wt. NaCl + 2% wt. CaCl_2_ (IS = 2.0 M)). Ethanol (purity: ≥95%), hydrochloric acid (quality level: 200), hydroxyethyl cellulose (HEC-10) (quality level: 100), 3-amniopropyl triethoxysilane (APTES) (purity: ≥97%), ethyl acetate (purity: ≥99.5%), acrylic acid (purity: ≥99%), and xylene (quality level: 100) (histology grade) were all purchased from Sigma Aldrich (St. Louis, MO, USA). Sodium bromide (purity: ≥99%) was purchased from Fisher Scientific (Waltham, MA, USA). Silica microspheres (8 nm low-polydispersity) were purchased from Fiber Optic, Inc. (New Bedford, MA, USA), and 2-amino-2-methylpropanesulfonate (AMPS) was provided by Lubrizol Corporation (Wickliffe, OH, USA). For experiments with silica and scale inhibitor in aqueous matrices, S807NM silica nanopowder was obtained from SkySpring Nanomaterials (Houston, TX, USA) and fumed silica nanoparticles (T30) were obtained from Wacker Chemie (Munich, Germany). These silica nanoparticles were used in all experiments with organic solvents. Fumed alumina nanoparticles (Alu130) were obtained from Evonik Industries (Essen, Germany). Flosperse MAS (FMAS), a commercial polyacrylamide scale inhibitor, was obtained from SNF Water Science (Andrézieux, France). Commercial asphaltene inhibitors used in this study included polyisobutylene succinic anhydride tetraethylenepentamine 1,8-napthalic anhydride (PIBSI-TEPA-NA), obtained from Nelson Brothers (Birmingham, AL, USA), PAO-3186 obtained from Baker Hughes (Houston, TX, USA), and SS637 obtained from SkySpring Nanomaterials (Houston, TX, USA).

### 2.2. Methods

#### 2.2.1. Nanoparticle Synthesis

The synthesis of nanoparticles designed to withstand high salinity and temperature followed the procedures presented in our previous work [48,49]. The nMag nanoparticles presented in Section 3.1 were synthesized as follows: five grams of Fe (II) acetate were dissolved in 50 mL of TEG. The mixture was purged with argon, then heated to 210 °C with a 20-minute ramp time, after which the temperature was held for 120 min with rapid stirring. The resulting nMag nanoparticles were then centrifuged, redispersed, coated with silica and APTES, and then grafted with AMPS. For the scale inhibition cases presented in Section 3.2, S807NM particles were mixed with Flosperse MAS in 500 mg/L NaCl for 24 h to allow for equilibrium adsorption of the polymer onto the nanoparticles (‘loading’ the nanoparticles). For the asphaltene inhibition cases presented in Section 3.3, fumed silica or fumed alumina was mixed with the specified commercial asphaltene inhibitor polymer in xylene for 24 h to allow for adsorption of the polymer onto the nanoparticles. The preparation procedure for these nanoparticle formulations is detailed in our previous work [50]. A summary table for the nanoparticle formulations used in each presented experiment is in Table S4. Prior to use, all nanoparticle formulations were monitored for 30 days for particle deposition or creaming to ensure that the formulations are colloidally stable.

#### 2.2.2. Rapid Formation Damage Screening Test Apparatus

**Figure 1:**
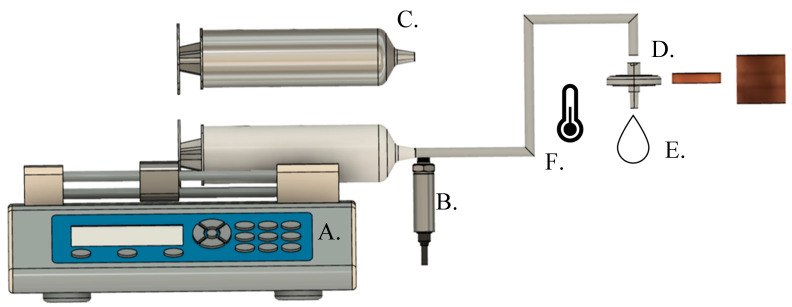
Schematic diagram of the formation damage test apparatus, which can be modified to allow for (**A**) flow rate adjustment, (**B**) transducers to monitor changes in pressure, (**C**) metal components (i.e., syringe/tubing) for organic mobile phase applications, (**D**) longer sandstone segments, (**E**) variations in solution composition (i.e., nanoparticle, polymers, dispersing fluid), and (**F**) heat tape for higher temperature testing and temperature control.

A formation damage screening test apparatus was developed to rapidly evaluate nanoparticle formulations for their mobility and potential for face-caking and formation damage over a range of experimental conditions (e.g., salinity, temperature, polymer addition). The apparatus consisted of a stainless steel, 2.5 cm diameter syringe filter holder (Model KS 25, Sterlitech Corporation, Kent, WA, USA) containing a Viton O-ring to seal the intact core discs (Figure 1). A syringe pump (Chemyx, Stafford, TX, USA) was connected to 1/8″ PTFE tubing with stainless steel Luer-lock connections, and a 3-way Hamilton valve was utilized to inject nanoparticle suspensions at a flow rate of 0.5 mL/min. This flow rate is consistent with those used in other nanoparticle-mediated enhanced oil recovery studies in similar Berea sandstone media [51,52]. Sandstone discs were cut from 2.5 cm diameter Berea sandstone cores with a wet tile saw (Black & Decker, Baltimore, MD, USA) to an approximate 2 mm thickness. To monitor pressure variations, a transducer (Chemyx, Stafford, TX, USA) was equipped to the system. The set-up allowed for pressure to be recorded up to a maximum of 390 PSI with ±0.1 PSI accuracy and records captured in 60 s increments. The system can undergo several modifications for a variety of needs, as depicted in the schematic (Figure 1). Table S3 provides further details regarding specific alterations to the systems made to test in organic solvents as opposed to water-based matrices. Prior to testing the apparatus for nanoparticle formations, control experiments were carried out at each experimental condition in the absence of nanoparticles to ensure that mobilization of naturally occurring matter, such as clay or inorganic matter, such as minerals, does not contribute to the pressure differential in the system. Control experiments confirmed that these processes are negligible and do not cause changes in permeability or porosity. For this work, to ensure method consistency, experiments that passed the screening test, that is, they did not indicate any signs of formation damage, were tested again to validate that conclusion.

#### 2.2.3. Core Flood Apparatus

The core flood apparatus set-up and procedures are consistent with those of our previously published works [49,52]. For conventional core flood experiments conducted with nMag, the intact sandstone cores (2.5 cm dia. × 7.5 cm length) were covered with high-strength (2-ton) epoxy (Ellsworth Adhesives, Germantown, WI, USA) along the length of the core to prohibit lateral flow through the sides. Following application, epoxy was allowed to cure for 24 h prior to any proceeding steps such as core saturation. Epoxy was only applied to the exterior surface of the sandstone cores and did not contact internal pore surfaces, and therefore, was not expected to alter intrinsic wettability or permeability. Wrap-tight tubing (1.45″ID before shrinking, McMaster-Carr, Elmhurt, IL, USA) was then placed around the middle of the epoxied intact sandstone, and heat was applied to tightly fit the wrap-tight tubing around the intact stone (Figure S1). High-strength epoxy was applied to fill the annular space between the epoxied sandstone core and the polycarbonate tube. Industrial-grade silicone sealant (DOWSIL™ 700, Dow Corning, Midland, MI, USA) was applied with a caulk gun and precision dispensing tip (Fisnar, Germantown, WI, USA) to fill the remaining annular space. The core holder set-up ensures flow uniformity within the sandstone cores. Each end of the polycarbonate tube containing the epoxied sandstone was fitted with a 1.25″ PVC compression male adapter (NDS, Lindsay, CA, USA), thick-wall PVC pipe fitting (McMaster-Carr), type 316 stainless steel threaded pipe fitting (McMaster-Carr), and type 316 stainless steel Yor-Lok tube fitting (McMaster-Carr, Elmhurt, IL, USA) in this order. The ends of the intact core system were equipped with 1/8″ Swagelok tube fittings with 316 stainless steel ferrules (Swagelok, Solon, OH, USA) to connect 1/8″ PTFE tubing to a female threaded opening, which was connected to 3-way valves (Hamilton Company, Reno, NV, USA). The core holder system is shown in Figure S2. For core experiments run at elevated temperatures, the column apparatus was heated with silicone heat tape (1″ wide × 2′ long, BriskHeat, Columbus, OH, USA) that was wrapped around the core apparatus and attached to a digital temperature controller (BriskHeat, Columbus, OH, USA).

#### 2.2.4. Core Flood Experiments

**Figure 2:**
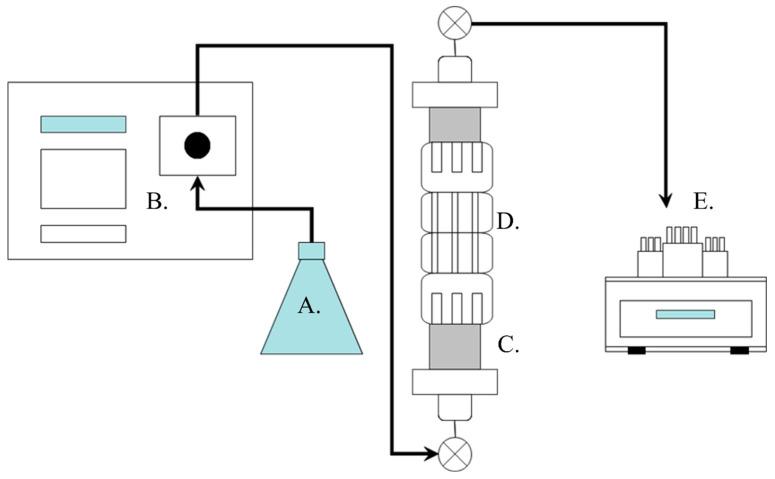
Schematic diagram of the experimental apparatus used for core flood experiments comprising (**A**) influent solution of nanoparticle suspension or background, (**B**) HPLC pump with injection pressure monitoring, (**C**) sandstone core holder, (**D**) heat band with temperature control, and (**E**) fraction collector.

After the core holder was assembled, the system was purged with carbon dioxide for 30 min to displace the air in the pore space. The system was then saturated with 10 pore volumes of a background solution (500 mg/L NaCl or API brine) at a volumetric flow rate of 1.0 mL/min using a Dynamix SD-200 pump (Varian Inc., Palo Alto, CA, USA). A nonreactive tracer test was conducted for each core by pumping ~3.5 pore volumes of 500 mg/L (if using 500 mg/L NaCl) or 2000 mg/L NaBr solution (if using API brine) into the system with a Series I HPLC Pump (Chrom Tech, Apple Valley, MN, USA), followed by an additional ~3.5 pore volumes of the background solution. Effluent samples were collected in 15 mL centrifuge tubes continuously with a CF-2 SpectraChrom fraction collector (Spectrum Laboratories, Rancho Dominguez, CA, USA). The core flood apparatus is depicted in Figure 2. The nMag suspensions were injected following the same procedure as the tracer test, with approximately 3 pore volumes of the nanoparticle suspension followed by an additional 3 pore volumes of the background solution injected at a pore-water velocity of 12 m/day (1.4 × 10^−4^ m/s). Photographs were taken of the core faces upon completion of each experiment. A staging area was set up and used for photographs to allow for consistency in factors including lighting, composition, and focus.

#### 2.2.5. Analytical Methods

Effluent samples collected during the non-reactive tracer tests were analyzed using an ion-selective bromide probe (Cole-Palmer, Vernon Hills, IL, USA). Calibration standards were prepared between 0 mM and 2 mM bromide, and effluent tracer samples were diluted accordingly. The resulting bromide effluent breakthrough curve data were fit with STANMOD CFITIM3 to determine the Peclet number and confirm the pore volume for the experiment [53] (Table S1). Effluent concentrations of nMag were analyzed with a Shimadzu UV-Vis 1800 spectrophotometer (Shimadzu Corporation, Kyoto, Japan). Calibration standards were prepared at concentrations between 0 and 1000 mg/L iron oxide and were measured at a wavelength of 600 nm. Representative calibration curves for the bromide probe and nMag UV-Vis spectroscopy are provided in Figures S3 and S4, respectively.

To verify nanoparticle deposition on the inlet face of the sandstone cores, images were processed with ImageJ (Version 1.54) software [54]. The images were uploaded, converted to 32-bit grayscale, and a single colour scheme was applied. Each face was analyzed with a colour histogram tool, and the number of pixels in each shade was normalized by the total number of pixels in the image. Additionally, heatmaps of nanoparticle deposition were created by uploading images of the sandstone disc faces, splitting the colour images into their respective RGB (red, green, blue) channels, and then applying a single colour scheme to the R channel for each face in the same experiment [55]. No manual thresholding was applied, and comparative analyses were performed only among images acquired under identical conditions and processed using identical settings. Image analysis was not utilized to quantify concentrations of nanoparticles deposited on the face of the discs. Instead, it served as a visual tool to confirm that signs of formation damage were in fact due to nanoparticle deposition. Numerical modelling was performed in Python (Version 3.11) using the pressure data to determine nanoparticle deposition rates. The model relates nanoparticle deposition and attachment with a decrease in porosity and subsequent pressure change as the decreased permeability inhibits flow. Nanoparticle deposition rates were fit with the use of optimization functions in SciPy (Version 1.11), minimizing the residual sum of squares between the model and experiment [56].

## 3. Results and Discussion

### 3.1. Salinity Case Study: Rapid Screening Test

**Figure 3:**
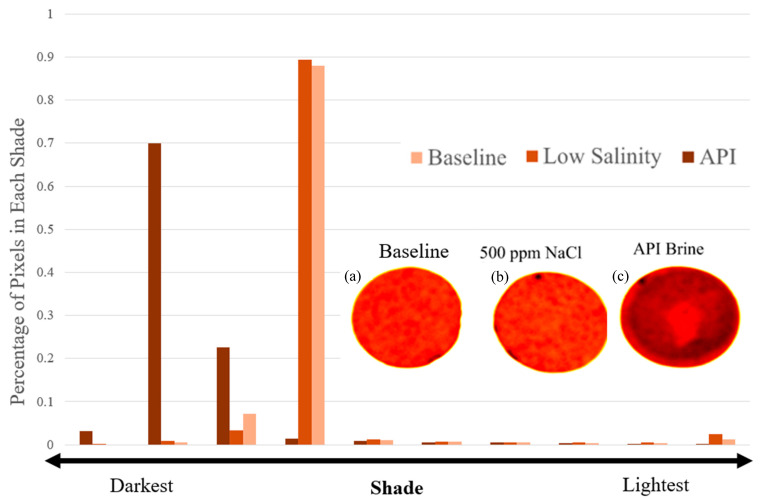
Processed images of rapid-test apparatus Berea sandstone discs: (**a**) baseline, (**b**) 500 ppm NaCl, (**c**) API brine showing colour shift due to high quantity of face-caking nanoparticles in API brine background.

It is well established that nanoparticles frequently exhibit decreased mobility in the presence of high salinity conditions due to nanoparticle aggregation, which can lead to greater particle attachment and physical straining [49,57,58]. For example, in high ionic strength API brine, which contains divalent cations (Ca^2+^), nanoparticle mobility in sandstone cores has been shown to be greatly reduced [59]. The presence of monovalent and divalent cations acts to compress the electric double layer (EDL) of metal oxide nanoparticles, which leads to particle aggregation [59,60,61]. Cation bridging between particles or between particles and the negatively charged mineral surfaces may also contribute to greater particle deposition in porous media [62]. This behaviour provides for a simple scenario to test the rapid screening device for formation damage as a function of ionic strength. For these tests, a clean 2 mm thick sandstone disc was placed in the formation damage test apparatus. For the first test, the disc was saturated with 500 mg/L NaCl, and then a 1,000 mg/L solution of nMag in 500 mg/L NaCl was injected through the disc at 0.5 mL/min. For the second test, the same procedure was repeated using the high salinity API brine solution in place of the 500 mg/L NaCl. At the termination of each screening test, the disc was removed and inspected for nanoparticle accumulation at the inlet (face-caking). Processed images of the inlet face of the sandstone for the control, low salinity (500 mg/L NaCl), and API brine solution tests are shown in Figure 3. Detailed colloidal functionalization of the nMag nanoparticles has been reported previously [48,49]. The screening tool is designed to directly capture the potential for face-caking and permeability reduction without the need to detail particle characterization.

**Figure 4:**
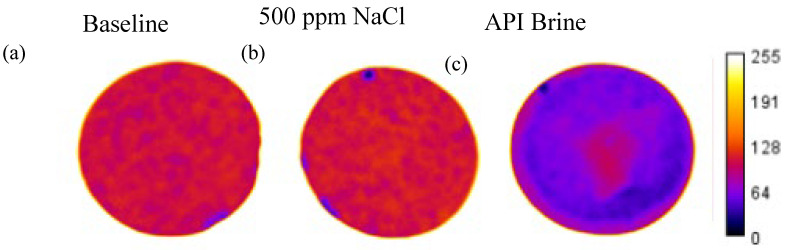
Colour intensity heatmap applied to Berea sandstone discs to map nanoparticle deposition due to changes in salinity conditions: (**a**) baseline, (**b**) 500 ppm NaCl, (**c**) API brine.

A single 10-shade colour scheme was applied to the three images of the discs, and then the number of pixels in each shade was quantified and normalized to the total number of pixels in each image (Figure 3). As the nMag suspension was dark brown in colour, the deposition of nanoparticles would make the disc appear darker than the baseline case. The low salinity case is nearly identical to the baseline, indicating minimal nanoparticle deposition on the face. Similarly, a heatmap can be applied to the disc images (Figure 4) after splitting each image into RGB channels and applying the same colour table to the same channel (in this case, R) for each image. The calibration bar represents the intensity of each pixel, where 255 is the most intense (white) and 0 is the least intense (black). The less intense colour of the disc from the API brine test is due to the deposition of nanoparticles. The image analysis approach provided results that were consistent with quantitative methods. For example, weighing the dried red discs before and after injection showed that there was no change in mass for the discs used as the baseline and with NaCl, while the disc associated with the API brine tests increased in weight due to deposited nanoparticles. Similarly, applying the UV method described in Section 2.2.5 and Figure S4 to the influent and effluent solutions, a measurable decrease in nanoparticle concentration was detected in the effluent solution for the API brine tests due to the mass deposited on the face of the sandstone.

#### Salinity Case Study: Core Flood Test

**Figure 5:**
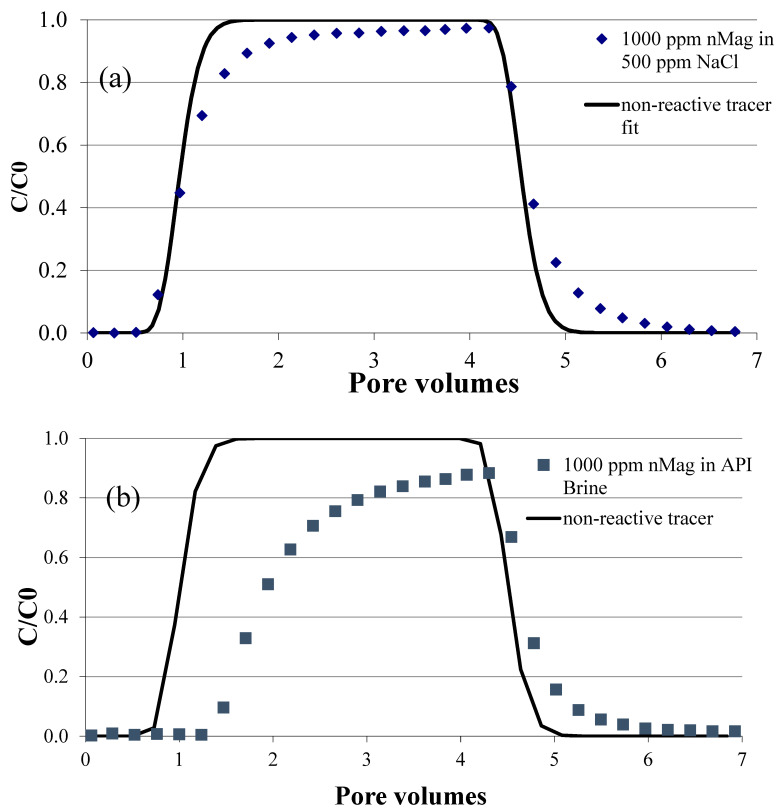
Core flood test results showing breakthrough of the non-reactive tracer and nMag for (**a**) 500 mg/L NaCl and (**b**) API brine. Note that the pulse width of the non-reactive tracer test was slightly smaller (i.e., ~3 pore volumes) in the 500 mg/L NaCl experiment.

**Figure 6:**
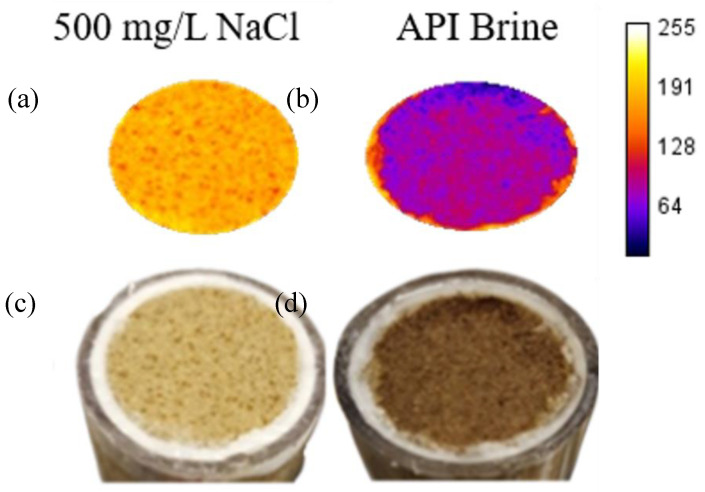
Intensity heatmap (**a**,**b**) and unprocessed images (**c**,**d**) of faces of sandstone cores for core flood tests with suspensions of 1000 mg/L nMag in either 500 mg/L NaCl (**a**,**c**) or API brine (**b**,**d**).

To validate the predictive capability of the rapid screening test, a core flood test was conducted with the nMag suspensions in each of the background solutions using the procedure described in Section 2.2.3. The volume average hydrodynamic diameters are consistent with those reported for these particles in our previous work and measured to be 148 nm in aqueous solution [49]. As shown in Figure 5a, nMag in the 500 mg/L NaCl solution exhibited breakthrough behaviour similar to that of the tracer, with greater than 95% of the injected nMag mass measured in the effluent samples. Table S1 presents the pulse widths and experimental results obtained from the tracers. These results indicate the high mobility of nMag through the sandstone core, consistent with the minimal accumulation of nMag on the disc under low salinity (500 mg/L NaCl) conditions. The observed decrease in mobility of nMag in the API brine was evident in the effluent breakthrough curve, which showed delayed breakthrough (i.e., >1 pore volume). In contrast to the low salinity case, only 63% of the injected nMag mass was detected in the effluent samples of the API brine core flood (Figure 5b). The inlet faces of the core flood experiments exhibited behaviour consistent with the rapid screening test apparatus, as shown in Figure 6, where the inlet face of the core flood conducted with API brine was coated with a thin layer of nMag. The image analysis in Figure 6 is consistent with quantitative weight and UV measurements as described in Section Salinity Case Study: Core Flood Test.

### 3.2. Nanoparticle Concentration Case Study: Rapid Screening Test

**Figure 7:**
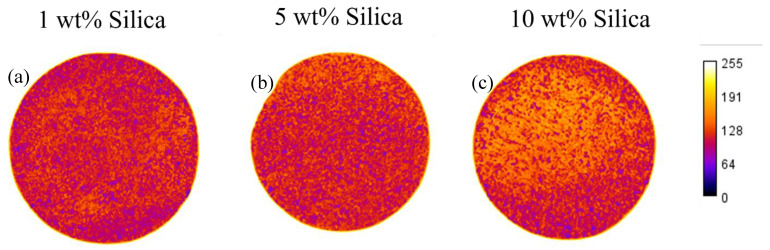
Heatmaps of colour intensity of Berea sandstone discs after injecting different silica nanoparticles at three concentrations (**a**) 1 wt.%, (**b**) 5 wt.%, (**c**) 10 wt.% with rapid screening apparatus.

A second application of the rapid screening apparatus is to determine the maximum concentration of nanoparticles that can be injected without causing formation damage or face-caking. To test this application of the device, the system was equipped with a transducer to measure pressure changes over time. Three concentrations (1, 5, and 10 wt.%) of silica nanoparticles used for scale inhibition were injected into the screening apparatus in a background solution of 500 mg/L NaCl. No pressure changes were observed during the injections of the solutions with 1 wt.% and 5 wt.% nanoparticles. However, when increasing to the 10 wt.% silica nanoparticle solution, a pressure increase of 55 PSI was observed. After completion of the tests, each disc was examined visually for nanoparticle deposition. Since these injected solutions were milky-white in colour, a disc with higher intensity readings would indicate nanoparticle deposition and face-caking. As shown in Figure 7, there was a shift to higher intensity colour due to nanoparticle deposition for the case with 10 wt% silica, in contrast to that of the lower concentrations.

### 3.3. Organic Solvent Case Study: Rapid Screening Test

To expand the versatility of the screening tool, several modifications were made to allow for organic solvents (e.g., xylene) to be used as the mobile phase. The plastic syringe and PTFE tubing were replaced with stainless steel components, and the core holder was extended to allow for the use of a 2 cm thick disc, in place of the original 2 mm thick disc. The increase in disc thickness was made at the request of the sponsor. In addition, heat tape was used to increase the temperature to 80 °C, which is more representative of reservoir conditions. Several nanoparticle formulations consisting of either silica or alumina nanoparticles mixed with various asphaltene inhibition polymers were tested to downselect (i.e., remove from consideration) formulations that may cause formation damage. With the implementation of the longer 2 cm thick disc, a simplified mathematical model was used to estimate the nanoparticle deposition rate for formulations that caused face-caking.

Here, the model assumed that as nanoparticles are deposited, the formation porosity decreases, and in turn reduces the permeability, which is related to the pressure changes that are monitored with the transducer. The fluid flow is assumed to be governed by Darcy’s law. which is coupled to permeability changes using the Kozeny-Carman (KC) model [63,64]. The core was divided into *N* equal sub-sections (N = 20) with the total pressure change (Δ*P*, Pa) following Darcy’s law where *μ* is the viscosity of the nanoparticle suspension (Pa-s), *Q* is the flow rate for the test (m^3^/s), Δ*x* is the length of each segment (m), *A* is the core cross-sectional area (m^2^), and *k_i_* is the segment *i* permeability (m^2^):

(1) $$∆P=\sum_{i=1}^{N}\frac{\mu Q∆x}{k_{i}A}$$

The permeability is assumed to be a function of porosity, as governed by the KC relationship between permeability and average grain diameter, d_p_ (m) and porosity (Equation (2)), where the porosity in each segment (ε_i_) changes due to the rate of nanoparticle deposition (k_dep_, 1/s). The nanoparticle mass concentration, C_i_ (-) in solution in a segment decreases with each time interval, ∆t (Equation (3)).

(2) $$k_{i}=\frac{d_{p}^{2}}{180}\frac{\varepsilon_{i}^{3}}{(1-\varepsilon_{i}{)}^{2}}$$

(3) $$\varepsilon_{i}\left(t+∆t\right)=\varepsilon_{i}\left(t\right)-k_{dep}C_{i}\left(t\right)∆t$$

The purpose of the permeability model is to provide a first-order, physically interpretable link between observed pressure increases and effective nanoparticle deposition during the screening tests. The model assumes homogeneous porosity reduction within discretized segments, constant effective particle size, and, for simplicity, associated with rapid screening, does not explicitly resolve localized inlet-scale heterogeneity associated with face-caking. Thus, the fitted deposition rate constant should be interpreted as an effective parameter representative of the bulk clogging behaviour rather than an intrinsic attachment rate. The parameters used in the model are summarized in Table S2. The pressure and time data were recorded from the transducer, and the deposition rate was fit as described in Section 2.2.5.

**Figure 8:**
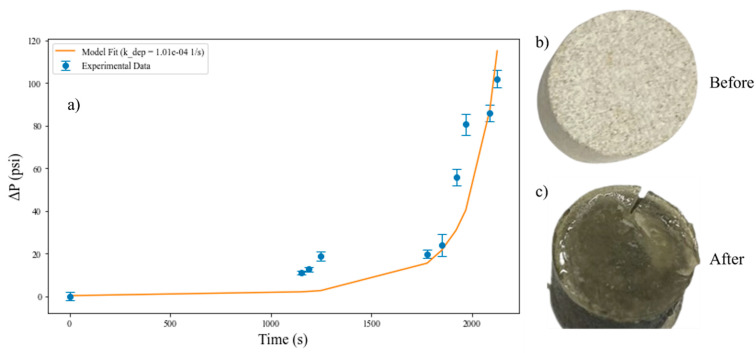
Rapid screening test results for 7 wt% T30 Silica + 28 wt% SS637 in xylene; (**a**) Experimental data and model predictions (r^2^ = 0.8) with a fitted deposition rate of 1.01 × 10^−4^ s^−1^, and (**b**) sandstone disc prior to test, and (**c**) sandstone disc after the test.

In the first test with a nanoparticle suspension composed of 7 wt.% T30 Silica nanoparticles and 28% SS637 asphaltene inhibitor polymer in xylene, the injection pressure increased rapidly after approximately 20 min of injection, and face-caking was clearly visible at the disc inlet (Figure 8). As the inlet clogged, the particles continued to deposit, and a gel formed at the inlet of the core.

Additional examples of organic solvent tests where face-caking was and was not observed are presented in the Supporting Information (Figures S5 and S6, respectively). For example, a 10 wt.% PIBSI-TEPA-NA + 10 wt.% silica suspension exhibited face-caking, while a solution with the same 10 wt.% PIBSI-TEPA-NA and 10 wt.% alumina in place of silica did not exhibit this behaviour. While both suspensions appeared stable prior to injection, the screening apparatus provided a quick method to gain insight into the settling behaviour under dynamic flow conditions, which is important to avoid formation damage. These tests provide further evidence of the utility of the disc apparatus for rapid screening of nanoparticle formulations.

## 4. Conclusions

Formation damage associated with nanoparticle injection is often dominated by near-inlet processes where aggregation and face-caking preferentially occur. The disc-based screening approach presented in this work is intentionally designed to interrogate these inlet-dominated mechanisms rather than to provide direct quantitative scaling to field-scale behaviour, as formulations that exhibit face-caking at the disc scale are likely to pose injectivity risks under core-flood and near-wellbore conditions. The tool is valuable in saving material costs and time, as it employs small sandstone discs as opposed to longer sandstone cores, and furthermore, the risk of formation damage can be determined on the order of magnitude of about 1 h per test. The device was shown to be useful for a variety of nanoparticle applications in both water and organic solvent-based formulations, indicating that the test allows for versatility in mobile phases. The apparatus is readily adaptable for elevated-temperature operation, as the organic solvent systems presented were tested at 80 °C. Temperature-dependent screening represents a valuable extension of the method for formulation-specific and site-specific evaluation. The presented case studies illustrate that the rapid test apparatus can be used to identify the salinity levels and injection concentrations for which formation damage is likely to occur. The rapid screening apparatus test results were found to be comparable to conventional core-flood tests. Injection pressure data can be used to estimate nanoparticle deposition rates, and a model was employed based on experimental data to confirm qualitative and quantitative observations. Both qualitative (i.e., visual inspection) and quantitative (i.e., pressure changes) information were used to validate the results of the screening tool with core flood tests. This disc apparatus has a wide range of potential applications where rapid down selection of nanoparticle formulations is advantageous, and could be used to evaluate the use of nanoparticles for characterization and recovery of unconventional oil and gas resources.

## Data Availability

The original contributions presented in this study are included in the article/Supplementary Material. Further inquiries can be directed to the corresponding author.

## Supplementary Materials

The following supporting information can be downloaded at: https://www.mdpi.com/article/10.3390/nano16070402/s1, Figure S1: Preparation of Berea sandstone core; Figure S2: Intact core system; Figure S3: Bromide calibration curve; Figure S4: nMag calibration curve; Figure S5: Face-caking images for 5 wt% silica + 10 wt% SS637; Figure S6: Face-caking images for 6 wt% alumina + 28 wt% PAO3186; Table S1: Experimental parameters for core flood tests; Table S2: Model parameters for Figure 8; Table S3: Testing apparatus parameters for aqueous and organic solvent systems; Table S4: Summary of nanoparticle formulations.
