# Supplementary material for: Rapid Screening Method to Assess Formation Damage During Injection of Metal Oxide Nanoparticles in Sandstone
Source: Nanomaterials (Basel). 2026 Mar 26;16(7):402. doi: 10.3390/nano16070402 (PMC13074735; doi:10.3390/nano16070402)
Supplement: Supplementary file 1 [file nanomaterials-16-00402-s001.zip › nanomaterials-4202590-supplementary.pdf]

nanomaterials

Supplemental Information

Rapid Screening Method to Assess Formation Damage During Injection of Metal Oxide Nanoparticles in Sandstone

Craig Klevan [1,†], Bonnie A. Marion [2,‡], Jae Jin Han [2,§], Taeyoung Chang [3], Shuhao Liu [4], Keith P. Johnston [3], Linda M. Abriola [1] and Kurt D. Pennell [1,*]

1 School of Engineering, Brown University, Providence, RI 02912, USA; craig.klevan@geosyntec.com (C.K.); linda_abriola@brown.edu (L.M.A.)
2 Department of Civil and Environmental Engineering, Tufts University, Medford, MA 02155, USA; bmarion@ftdsolutions.net (B.A.M.); jjlisahan@gmail.com (J.J.H.)
3 McKetta Department of Chemical Engineering, The University of Texas at Austin, Austin, TX 78712, USA; taeyoung@utexas.edu (T.C.); kpj@che.utexas.edu (K.P.J.)
4 The Jasper Department of Chemical Engineering, The University of Texas at Tyler, Tyler, TX 75799, USA; sliu@uttyler.edu
* Correspondence: kurt_pennell@brown.edu
† Current address: Geosyntec Consultants, Columbia, MD 21044, USA.
‡ Current address: FTD Solutions Inc., Colorado Springs, CO 80919, USA.
§ Current address: Graduate School, Boston, MA 02138, USA.

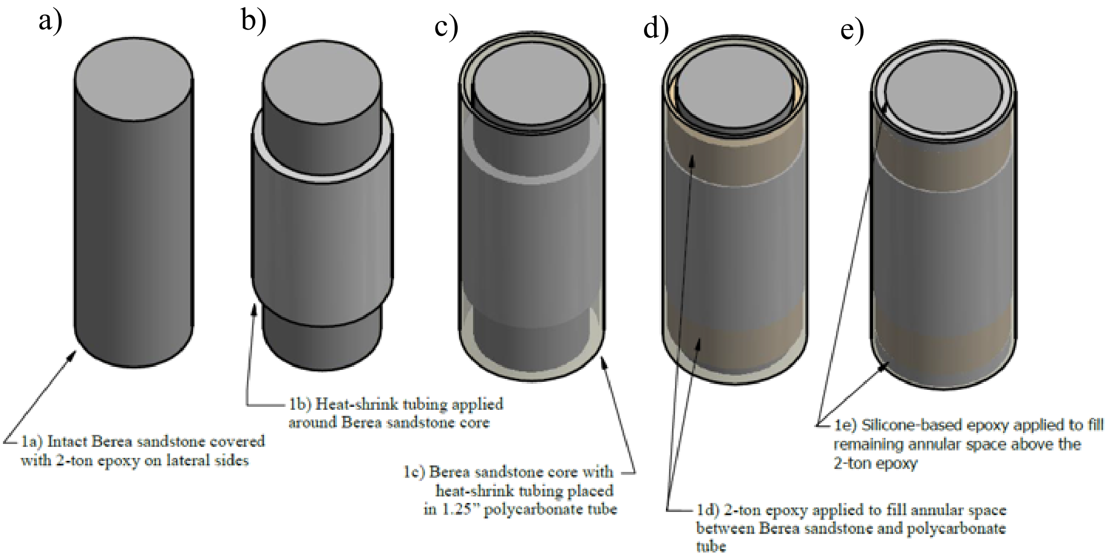

Figure S1. Preparation of Berea sandstone core. Intact Berea sandstone (a) covered with 2-ton epoxy on lateral sides, (b) heat shrink tubing placed applied around Berea sandstone core, (c) Berea sandstone with heat shrink tubing placed in 1.25" polycarbonate tube, (d) epoxy applied to fill annular space between Berea sandstone core and polycarbonate tube, and (e) Silicone-based epoxy applied to fill remaining annular space above the 2-ton epoxy.

 https://doi.org/10.3390/nano16070402

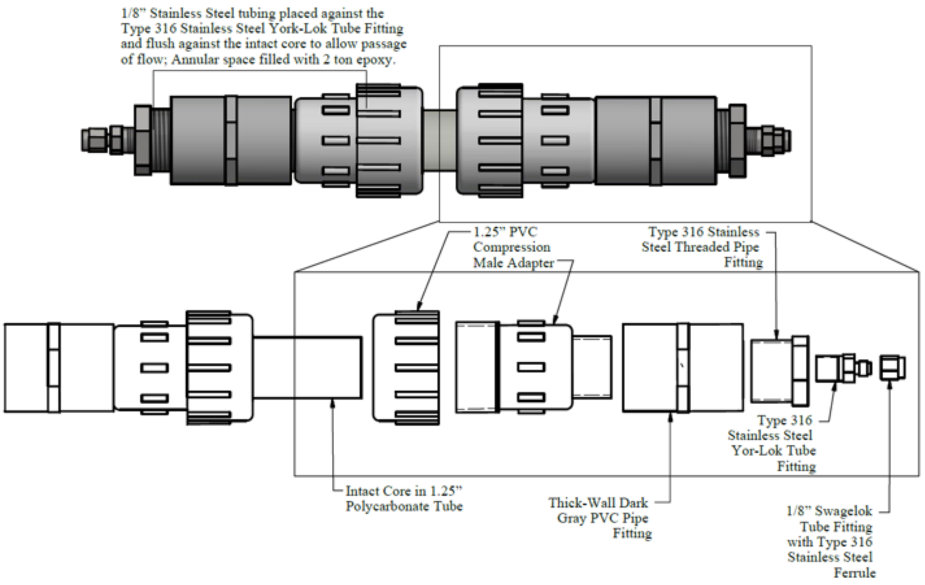

Figure S2. Intact core system. Intact core system with fitting parts from bottom to top: 1.25″ PVC compression male adapter, thick-wall PVC pipe fitting, Type 316 stainless steel threaded pipe fitting, Type 316 stainless steel Yor-lock tube fitting, 1/8″ Swagelok tube fitting with Type 316 stainless steel ferrule to connect 1/8″ plastic tubing to a female threaded opening, and 3-way Hamilton valve. High strength epoxy fills the annular space around the 1/8″ stainless steel pipe.

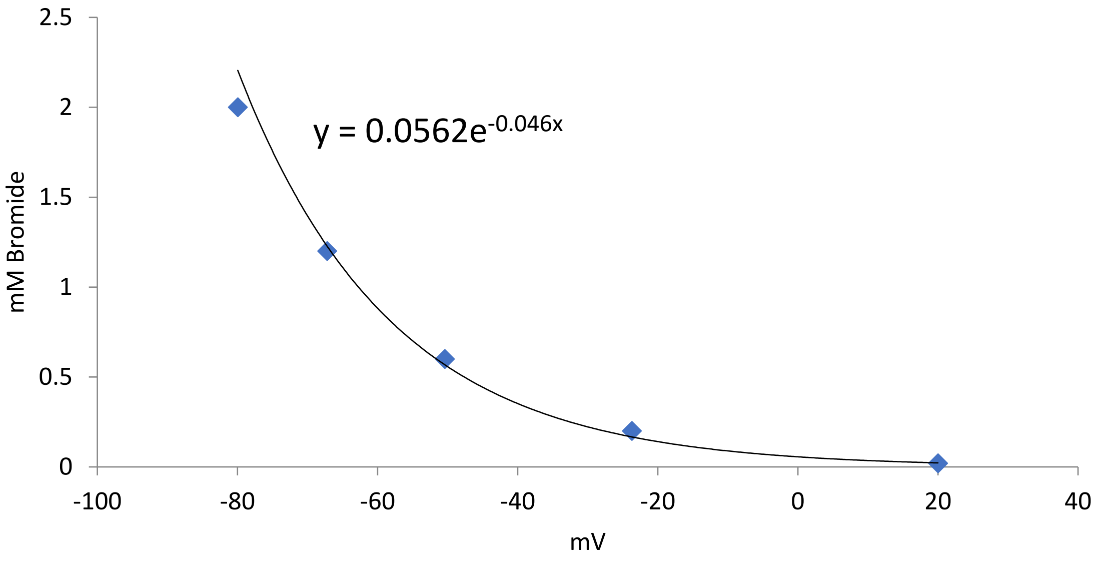

Figure S3. Bromide calibration curve. Representative calibration curve used for bromide ion analysis obtained from bromide probe measurement of tracer test samples.

https://doi.org/10.3390/nano16070402

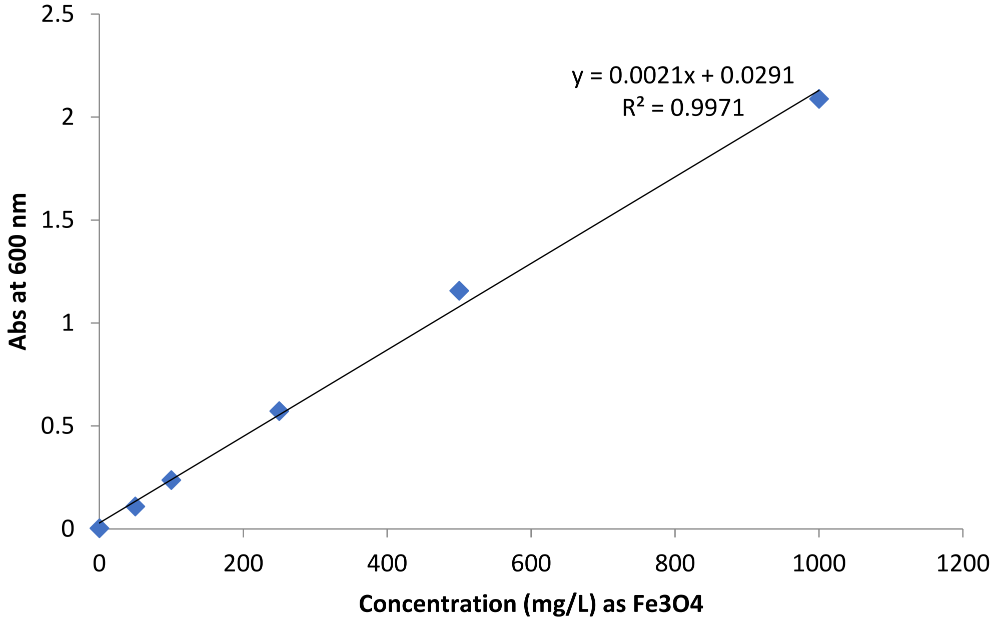

Figure S4. nMag calibration curve. Representative calibration curve used for effluent nMag concentration analysis obtained from UV-Vis Spectroscopy at 600 nm wavelength.

Table S1. Experimental parameters for core flood tests. Experimental parameters and results for the core flood test results showing breakthrough of the non-reactive tracer and nMag for a) 500 mg/L NaCl and b) API brine shown in Figure 5.

	Experimental Parameters					Experimental Results		
Fig.	nMag (mg/L)	Background Solution	Temp. °C	Pulse Width (PV)	Core Material	Pe	Rf	nMag BT %
5a	1000	API Brine	22	3.45	Berea	60.1	1.09	62.6
5b	1000	500 mg/L NaCl	22	3.7	Berea	33.6	1.02	96

Table S2. Model parameters for Figure 8. Model parameters for the rapid screening test for 7 wt% T30 Silica + 28 wt% SS637 in xylene shown in Figure 8.

Parameters	Symbol	Unit	Value	Method Obtained
Length	L	m	0.025	Measured
Area	A	m^2	5.0E-4	Measured
Flow Rate	Q	m^3/s	8.33E-9	Measured
Dynamic Viscosity	μ	Pa-s	0.6E-3	Measured
Initial Porosity	ε_i	(-)	0.23	Given by supplier
Average grain diameter	d_p	m	200E-6	Given by supplier
Number of segments	N	(-)	20	User defined
Time	t	s	Experiment time	Measured
Pressure	P	Pa	Transducer P	Continuously Measured

https://doi.org/10.3390/nano16070402

Table S3. Testing apparatus parameters for aqueous and organic solvent systems. System alterations for testing systems with nanoparticle suspensions in water or nanoparticle suspension in organic solvent.

Apparatus Component	Aqueous System	Organic Solvent System
Syringe Pump	Chemyx Fusion 100 High-Precision Syringe Pump (Chemyx Inc., Stafford, TX.)	Chemyx Fusion 6000X High Pressure Syringe Pump (Chemyx Inc., Stafford, TX.)
Syringe	60 mL Becton Dickinson syringe, polypropylene barrel, polyethylene plunger	100 mL Chemyx stainless, steel barrel and plunger with 2 oil resistant Viton® O-Rings
Tubing	Polytetrafluoroethylene (PTFE)	316 Stainless Steel

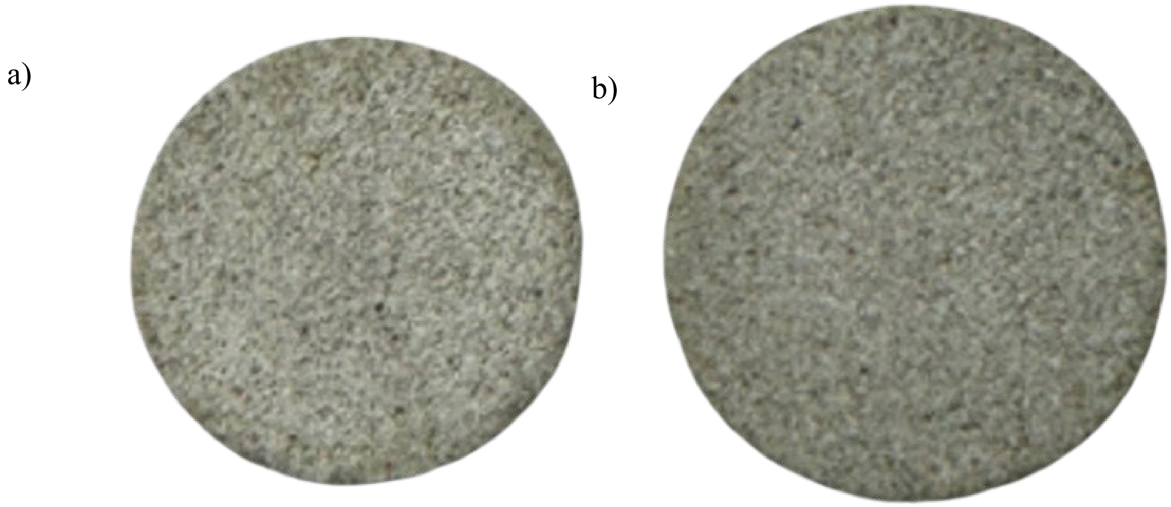

Figure S5. Face-caking images for 5 wt% silica + 10 wt% SS637. Face caking images from Section 3.3. No particle deposition or pressure changes were observed between a) before and b) after nanoparticle (5 wt% silica + 10 wt% SS637) injection.

a) b)

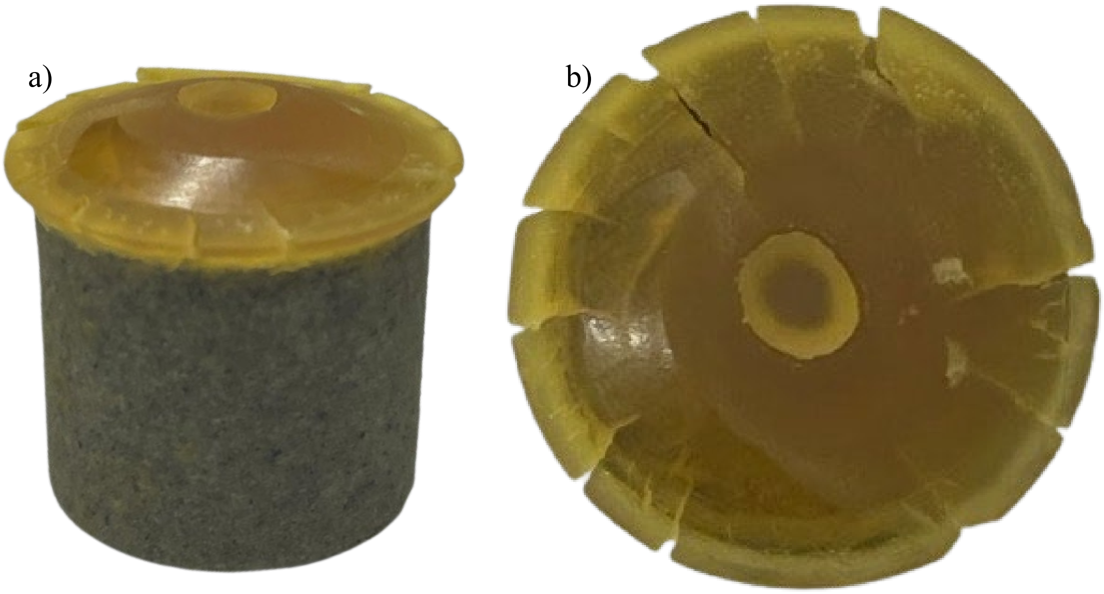

Figure S6. Face-caking images for 6 wt% alumina + 28 wt% PAO3186. Face-caking test using set up in Section 3.3 of manuscript. Nanoparticle (6 wt% alumina + 28 wt% PAO3186) resulted in extreme particle deposition as seen from a) side view and b) top view and large pressure jump.

Table S4. Summary of nanoparticle formulations. Summary table of nanoparticle formulations in each experiment presented.

Figure	Nanoparticle Formulation	Background Matrix
3b	1,000 mg/L nMag grafted with AMPS	500 mg/L NaCl
3c	1,000 mg/L nMag grafted with AMPS	API Brine
4b	1,000 mg/L nMag grafted with AMPS	500 mg/L NaCl
4c	1,000 mg/L nMag grafted with AMPS	API Brine
5a	1,000 mg/L nMag grafted with AMPS	500 mg/L NaCl
5b	1,000 mg/L nMag grafted with AMPS	API Brine
6a/c	1,000 mg/L nMag grafted with AMPS	500 mg/L NaCl
6b/d	1,000 mg/L nMag grafted with AMPS	API Brine
7a	1 wt% silica (S807NM) + 1 wt% FMAS	500 mg/L NaCl
7b	5 wt% silica (S807NM) + 5 wt% FMAS	500 mg/L NaCl
7c	10 wt% silica (S807NM) + 10 wt% FMAS	500 mg/L NaCl
8	7 wt% silica (T30) + 28 wt% SS637	Xylene
S5	5 wt% silica (T30) + 10 wt% SS637	Xylene
S6	6 wt% alumina + 28 wt% PAO3186	Xylene

https://doi.org/10.3390/nano16070402
